# Imaging Techniques for Meniscal Vasculature: A Systematic Review of Clinical and Translational Applications

**DOI:** 10.3390/jcm13226787

**Published:** 2024-11-11

**Authors:** Federica Orellana, Raluca-Ana-Maria Barna, Camilla Giulia Calastra, Annapaola Parrilli

**Affiliations:** 1 Center for X-Ray Analytics, Empa-Swiss Federal Laboratories for Materials Science and Technology, Überlandstrasse 129, 8600 Dübendorf, Switzerland; 2 Faculty of Science and Medicine, University of Fribourg, 1700 Fribourg, Switzerland; 3 Faculty of Medicine, University of Bern, 3012 Bern, Switzerland; 4 Spine Biomechanics, Balgrist University Hospital, 8008 Zurich, Switzerland

**Keywords:** meniscus, vascular network, imaging, 3D imaging, clinical application, translational application, animal models

## Abstract

**Purpose**: The focus of this review is on the imaging techniques used to visualize the meniscal vascular network and arteries in clinical, human ex vivo, and animal model applications. For this purpose, research articles from the past decade that have imaged the vascular network of the meniscus and/or the genicular and popliteal arteries were identified according to established PRISMA statement standards. **Methods**: Various imaging modalities, including magnetic resonance imaging, micro-computed tomography, and optical and fluorescence microscopy, were included and compared based on the type of visualization, imaging resolution, and range of vessel size detection. These imaging modalities were evaluated based on the outcomes of interest, including diagnostic accuracy in identifying the meniscal vasculature and associated pathologies, clinical applications to guide surgical decisions, and translational applications contributing to the research and development of new therapies and the understanding of meniscal physiology and pathology. **Results**: The analysis conducted in this study highlights the importance of imaging resolution and visualization type in accurately depicting the complex microvasculature of the meniscus with high precision and detail. **Conclusions**: This review underscores the necessity for high-resolution 3D imaging techniques to comprehensively understand the meniscal vascular network and enhance surgical approaches and treatment options for meniscal lesions and pathologies.

## 1. Introduction

The vascular network of the meniscus is crucial for its healing potential and regenerative capacity [1]. Indeed, in case of a tear, the vascularized regions of the meniscus have the potential to self-repair due to the formation of a fibrin clot that acts as a scaffold for neovascularization and attracts oxygen, nutrients, and essential factors to repair the lesion [2]. In contrast, tears in the avascularized zones have limited regenerative capacity and if left untreated can lead to the onset of osteoarthritis (OA) [2,3]. Based on the degree of vascular penetration, the meniscus is divided into four distinct vascularized circumferential zones. From the outer to the inner tissue, they are defined as the perimeniscal (PM) or zone 0, the red-red (RR) or zone 1, the red-white (RW) or zone 2, and the white-white (WW) or zone 3 [4,5]. Due to the high prevalence of meniscal tears, which are the most common knee injury, leading to approximately 61 meniscectomies per 100,000 patients annually [6], a detailed study on the meniscal vascular network is of paramount importance.

In 1982, Arnoczky and Warren conducted the first detailed study on the meniscal vascular network using two-dimensional (2D) optical microscopy and tissue-clearing techniques [4]. They discovered that the lateral and medial genicular arteries supply the meniscus by forming a perimeniscal capillary plexus (PCP) within the synovial and capsular tissues of the knee [4]. Optical microscopy, a well-established imaging technique, has been widely used to visualize the structure of biological samples in histological sections with high resolution. For this reason, it has been used extensively in both human and animal model ex vivo studies to identify even the smallest vascular structure of the lateral and medial meniscus [7,8,9,10,11,12,13]. However, due to the destructiveness of histological sample preparation, three-dimensional (3D) imaging techniques, such as micro-computed tomography (micro-CT) and 3D microscopy in general, are increasingly being used to capture the 3D complexity of the meniscal vascular network without compromising tissue integrity [10,14,15,16]. Clinically, most studies focus on imaging the arteries that directly nourish the meniscus, namely the genicular and popliteal arteries. For this purpose, magnetic resonance imaging (MRI) is often employed to map the location of these arteries within the knee joint, facilitating the development of patient-specific treatment strategies [17,18,19,20,21,22].

Despite the significance of the meniscal vascular network, there is, to our knowledge, no systematic review investigating the imaging modalities for meniscal blood vessel investigation. Therefore, the aim of this study is to evaluate the articles published in the past 10 years that have imaged and investigated the meniscal vascular network and/or the genicular and popliteal arteries. This review emphasizes the imaging techniques used in the analyzed studies, highlighting the importance of image resolution and 2D/3D visualization in distinguishing vascular network structures. Relevant studies providing clinical or translational insights using human samples and those exploring meniscal vascularity in animal models were assessed.

## 2. Materials and Methods

### 2.1. Eligibility Criteria

In this systematic review, we collected and included articles based on key parameters such as Population (P), Intervention (I), Comparison (C), and Outcomes (O), which together form the PICO framework. In detail, our key PICO parameters can be described as the following:

- P: Human patients with meniscal injuries or conditions requiring evaluation of meniscal vasculature. This population may include individuals who have experienced knee trauma, are recovering from meniscal surgery, or have conditions that affect meniscal perfusion. In addition, cadaveric studies and animal models are included specifically for the translational research component, providing insights into the meniscal vasculature and its role in injury and healing that can be applied to human patients.
- I: Imaging techniques used to visualize and evaluate the meniscal vasculature.
- C: Alternative or traditional imaging modalities that do not specifically focus on the meniscal vasculature. These may include standard meniscal imaging without vascular assessment, surgical or arthroscopic procedures without advanced vascular imaging, and lack of specific vascular imaging.
- O: Outcomes of interest include the following
  1. Diagnostic accuracy: The effectiveness of imaging techniques to accurately identify the meniscal vasculature and associated pathologies.
  2. Clinical applications: The role of these imaging modalities in guiding clinical decisions, such as surgical intervention or postoperative healing monitoring.
  3. Translational Applications: How these imaging modalities contribute to the research and development of new therapies or the understanding of meniscal physiology and pathology.

The imaging modalities include MRI, Ultrasound imaging, Contrast-Enhanced Computed tomography (CECT), Arthrography, Micro-CT, Microscopy, and other emerging or advanced techniques that allow visualization of the meniscal vasculature.

We excluded reviews, editorials, commentaries, conference abstracts, studies published in languages other than English, and articles that did not describe imaging techniques, as well as in vitro studies.

### 2.2. Search Strategy

**Figure 1:**
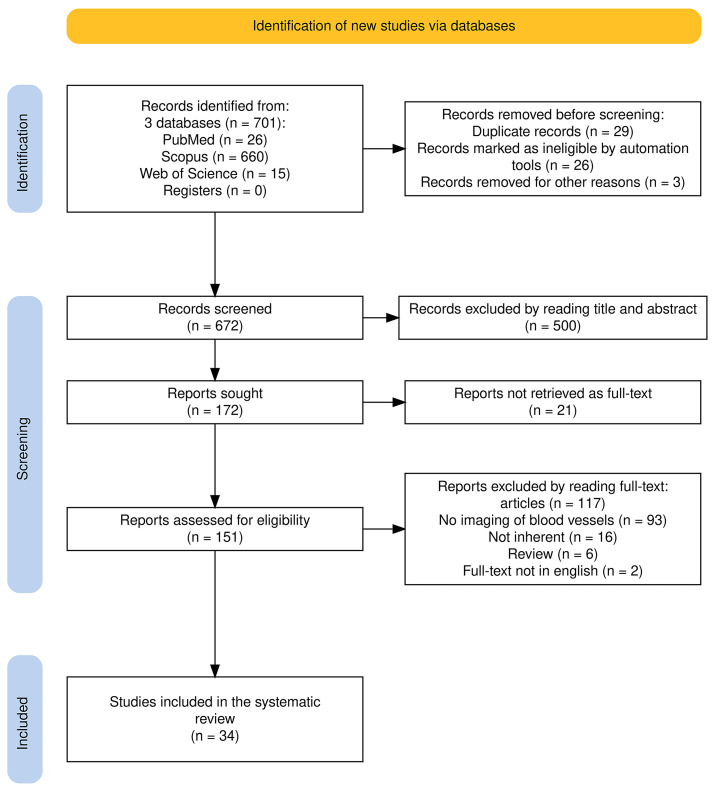
Schematic representation of the research strategy.

The systematic search was conducted on 9 August 2024, covering research published over the past decade (between 9 August 2014 and 9 August 2024). The search was performed according to the Preferred Reporting Items for Systematic Reviews and Meta-Analyses (PRISMA) guidelines across three databases: PubMed, Scopus, and Web of Knowledge (accessed on 9 August 2024), limiting the search to original articles in English. Using Boolean operators, the following string was applied: ((“meniscus” OR “menisci” OR “meniscal”) AND (“blood vessel” OR “blood supply” OR “vasculature” OR “microvasculature”) AND “imaging”). The articles were submitted to a public reference manager to eliminate duplicates and manage the references (Zotero version 7.0.1). Two reviewers (F.O. and A.P.) screened relevant articles using the title and abstract. The included full-text articles were then reviewed by all the authors, and any disagreement was resolved through discussion among the authors. A schematic representation of the studies search is given in Figure 1.

### 2.3. Extracted Data and Key Findings

The following information from each included paper was extracted and organized into Table 1 to summarize the evidence reported in each study: (a) Clinical/Animal model/Human ex vivo; (b) Imaging modality; (c) 2D or 3D visualization; (d) Imaging resolution; (e) Range of vessel size detection; (f) Evaluations; (g) Main Results; (h) Imaging Impact; and (i) References (Ref).

## 3. Results

### 3.1. Clinical Analysis

Clinical data on meniscal vascularization were analyzed in fifteen articles. Out of these, ten articles [17,18,19,20,21,22,26,28,30,31] employed MRI to investigate perfusion changes in meniscal disorders. The remaining five studies [23,24,25,27,29] utilized alternative imaging modalities.

Among these clinical studies, four articles [19,25,27,29] specifically evaluated meniscal vascularization. For instance, Kamimura [25] demonstrated that indocyanine green (ICG) fluorescence-guided knee arthroscopy enables real-time evaluation of vascularity and perfusion in knee structures, including the meniscus. In contrast, Guo et al. [19] explored whether intravoxel incoherent motion diffusion-weighted imaging could depict microcirculation within the meniscus. They discovered that the perfusion fraction is lower in the red zone of torn meniscus horns compared to normal ones. However, the pseudoand real-diffusion coefficients did not exhibit statistically significant differences among the groups. Furthermore, van Schie et al. [29] confirmed that meniscal vascularization could be visualized in real-time using near-infrared (NIR) fluorescence imaging with ICG, with vascularization observed in 75% of patients, showing similar extents (median vascularization of 13% with NIR fluorescence imaging versus 15% with ICG). Additionally, Poboży et al. [27] found that ultrasonography could detect major blood vessels and serve as evidence of ongoing remodeling of blood vessels within meniscal implants.

Two studies centered on the thickness of the popliteal artery wall as observed through MRI [30,31]. The earlier work by Wang et al. (2015) [30] associated popliteal artery wall thickness and tibial cartilage loss over two years. In a more recent follow-up study (2023) [31], the same group reported an association between increased popliteal artery wall thickness and progression in knee OA.

Other studies evaluated additional vascular and structural features of the knee using several imaging techniques. Choi et al. [18] used MRI to quantify bone marrow lesions and meniscal injuries in osteoarthritis patients concerning genicular artery embolization, revealing that larger bone marrow lesions and severe meniscal injuries indicated poor responses to the procedure. In another study, Bagla et al. [23] evaluated the effects of embolization on hypervascular genicular arteries using digital subtraction angiography, identifying a hypervascular “blush” over the medial inferior aspect of the knee from the superior medial genicular artery branches and demonstrating post-embolization “pruning” of the hypervascular synovium, while the parent genicular artery remained patent.

Further research on vascular malformations evaluated intra-articular knee hemangiomas through ultrasonography and T2-weighted MRI [28]. They found a lobulated, multi-septate hyperintense lesion with T2-hypointense septae, suggesting a slow-flow vascular malformation in Hoffa’s fat pad. Doppler ultrasound revealed small hypoechoic spaces with venous vascularity, providing a non-invasive option for identifying such vascular abnormalities.

Several articles (five out of fifteen) [20,21,22,24,26] focused on the anatomical evaluation of the popliteal and genicular vasculature around the knee joint, emphasizing distances from critical structures and the influence of patient characteristics or knee conditions. For example, Park et al. [21] assessed the diameter and height of the inferior lateral genicular artery, relative to the meniscocapsular junction across different meniscal zones. Their findings indicated that the inferior lateral genicular artery is positioned closest to the meniscocapsular junction in the middle zone, highlighting the importance of careful navigation during surgical procedures. Alternatively, Schachne et al. [22] explored the spatial relationship between the posterior horn of the lateral meniscus and the popliteal vasculature, noting that the distance progressively increases with growth and varies based on individual characteristics such as height, weight, and skeletal maturity. This spatial evaluation using MRI enabled the determination of the appropriate meniscal suture technique and minimized the risk of intraoperative vascular compromise during meniscal repairs. Nishimura et al. [26] examined the position of the popliteal artery in relation to surgical portals during knee flexion and extension, identifying the safe position of the artery for all-inside meniscal suturing. They suggested that all-inside meniscus suturing from the anteromedial portal is safest in a figure-of-four knee position and that repairs should be prioritized before anterior cruciate ligament (ACL) reconstruction to ensure optimal vascular safety and access. Keyurapan et al. [20] presented a detailed anatomical map of the neurovascular bundle, including the popliteal artery, vein, and tibial nerve, relative to posterior knee structures in various knee positions. They show that such structures shift posterolaterally during knee flexion, which is a critical result for surgeons performing procedures on the posterior knee to avoid inadvertent damage. Ezamin et al. [24] compared the distance between the popliteal artery and the tibial plateau in healthy and osteoarthritic knees, finding no significant correlation between popliteal artery proximity and OA severity, gender, or age. The results suggest that osteoarthritic changes may not impact vascular positioning. In addition, the findings underline the advantages of using CT angiography (CTA) for reliable and consistent measurements, even considering the associated risks of contrast media and radiation.

The study by Biolatto et al. [17] examined the risks to neurovascular structures during bicortical fixation of the tibial tubercle, identifying regions where the popliteal artery is at a higher risk of injury. This research aligns with other anatomical and imagingbased studies [21,22], which map out critical vascular distances around the knee joint and emphasize the variability of these distances based on patient characteristics and meniscal zones, while many articles [20,26] focus on understanding the spatial relationships of the popliteal artery to enhance surgical safety during procedures like meniscal repair or ACL reconstruction, Biolatto’s study narrows its focus to a specific surgical intervention—tibial tubercle screw fixation [17].

### 3.2. *Ex Vivo* Human Analysis

Human ex vivo samples have been employed in twelve of the eligible publications considered in this study (35.3%) [7,9,10,12,13,16,32,33,34,35,36,37]. Six of these studies used 2D histological evaluations to gain deeper insights into the meniscal microstructure, vascular organization, and associated cellularity [7,9,10,12,13,35]. The information obtained enabled comparisons of blood vessel distribution between different meniscal zones [10] and among various genders of the patients [7]. Furthermore, histology was employed to evaluate age differences and the effect of the aging process on microvascular density. In a study by Michel et al. [12], it was noted that vascular density declined with advancing age, and no vessel formations were observed in RW and WW zones after adolescence. In another study, histological analysis has been used to evaluate the consequences of discoid lateral meniscus in young patients, revealing an absence of blood vessels in the inner part of the meniscus in all cases except the 18-year-old [9].

Three studies investigated how pathological conditions of the knee joint associated with the progression of OA impact the blood vessel distribution within the meniscus. Wang et al. [13] utilized histology to microscopically grade the degenerative state of the meniscus in relation to OA. In their study, the distribution of blood vessels was associated with a tree-like formation of collagen fibers within the meniscus. The number of blood vessels along this structure decreased as degeneration advanced. When comparing osteoarthritis with rheumatoid arthritis, Fuhrmann et al. [35] observed that overall vascular density remained similar between patients from both groups. Additionally, microenvironmental variations in degenerative menisci were confirmed through immunofluorescence imaging, which revealed the presence of immune cells surrounding the blood vessels [34].

Contrast-enhanced MRI was used to assess meniscal perfusion in neonatal and adult cadaveric knees, revealing greater perfusion in the peripheral zone compared to the inner region, with higher overall blood flow in younger menisci [36]. MRI was also used to evaluate the risk of iatrogenic neurovascular injury during lateral meniscus repair, defining specific danger zones [33]. Similarly, vascular risk was evaluated in patients undergoing realignment osteotomies by three-dimensionally studying cadaveric knee vascularization using contrast-enhanced clinical CT [32]. The arrangement of the saphenous nerve branches and the popliteal neurovascular bundle in cadaveric knees was also analyzed using plastinated cross-sections, aiming to identify a low-risk configuration for the posteromedial knee portal [37].

A recently published study employed CECT to visualize and quantitatively analyze the meniscal vascular network [16]. In the study, the highest vascular volume was found in the peripheral PM zone, while the mid-posterior radial portion showed the lowest vascular contribution. Additionally, the vascular segments in the PM zone exhibited a different diameter compared to the other circumferential zones.

### 3.3. Animal Models Analysis

In this systematic review, seven eligible scientific articles, representing 20.6% of all included studies, utilized animal models to examine the vascular network of the meniscus and knee joint [8,11,14,15,38,39,40]. The majority of the studies utilized pigs (three out of seven) [15,38,39]. The remaining studies included one each for bovines [14], horses [11], and mice [40]. Additionally, one study employed both bovine and rabbit models [8], contributing to the total count of seven studies in the animal models group. Contrary to the clinical and human ex vivo articles, all the animal model studies focused solely on the meniscal vascular network [8,11,14,15,38,39,40], rather than the main arteries supplying the meniscus, such as the genicular and popliteal arteries.

Two recently published papers employed 3D imaging techniques to study the meniscal vascular network and quantify its vascular volume [15,40]. Light sheet microscopy, in combination with tissue clearing, was utilized by Sheng and collaborators to analyze in 3D the meniscal vascular network of transgenic mice. However, their findings revealed no significant differences in vascularization between the circumferential zones of the meniscus. Significant differences were found in the radial meniscal regions and between the medial and lateral meniscus. The anterior zone was found to be more vascularized than the central and posterior regions, and the medial meniscus more vascularized than the lateral [40].

In another study, Karjalainen et al. utilized micro-CT to analyze the vascular network of neonatal and adult pigs at a high resolution of 3.3 µm [15]. Their quantitative analysis revealed that neonatal menisci have a higher number of vascular segments, with more branching points and greater tortuosity, while adult menisci are characterized by fewer, but thicker, blood vessels. The age- and zone-related vascularization of the meniscus was also examined in horses using optical microscopy on histological slides [11]. Similarly to humans, the vascularization of equine menisci decreases with age, indicating that horses represent an ideal model for studying the healing potential of the meniscus [11].

In order to understand the meniscal response to injury and its biomechanical functions, another research group investigated the vascular network of rabbits and bovine menisci in relation to their structure and biochemical composition [8,14]. They employed optical microscopy, OPT, and second-harmonic-generation microscopy to visualize that blood vessels within the meniscus and its insertional roots are found adjacent to collagen fibers and proteoglycans (PGs)-rich regions [8].

Additionally, a new ICG fluorescence-guided knee arthroscopy method was investigated for its potential clinical use to study case-specific meniscal vascularization [38]. To evaluate this method, the author assessed the vasculature in both healthy and injured pig menisci. However, no fluorescence signal was detected in the meniscus [38]. In another study, researchers used pig animals to investigate transport mechanisms through blood vessels in the marrow cavity, bone, cartilage, and meniscus using fluorescence microscopy [39].

## 4. Discussion

**Figure 2:**
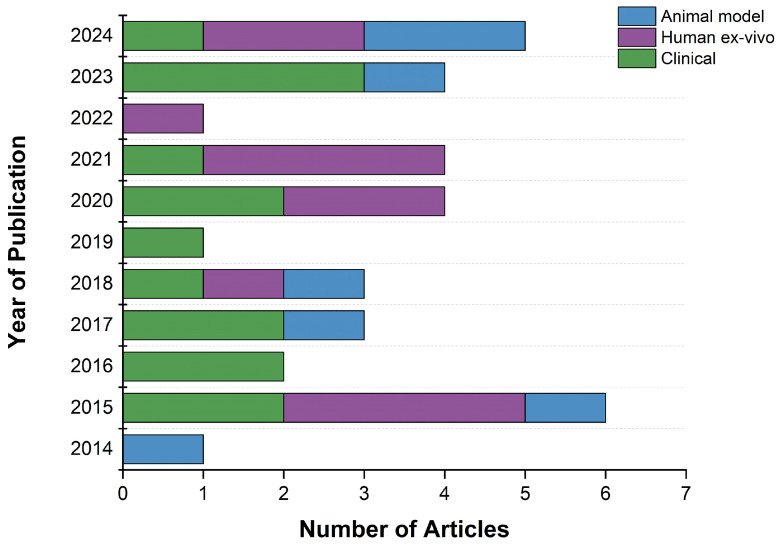
Trend in published articles by year and study type.

In this systematic review, the articles published over the last ten years that focus on imaging the meniscal vascular network and arteries were investigated. Every year at least one article was published, with the lowest number of publications in 2014, 2019, and 2022 (*n* = 1 per year) (Figure 2). In contrast, in 2015 and 2024 six and five publications, respectively, were found, including all the study types (animal model, human ex vivo, and clinical) Figure 2. This increase in the number of articles published in 2024 indicates the growing interest of the scientific community in imaging the meniscal vascular network and highlights the necessity to better understand this network using human ex vivo samples and animal models, with the goal of improving clinical strategies.

**Figure 3:**
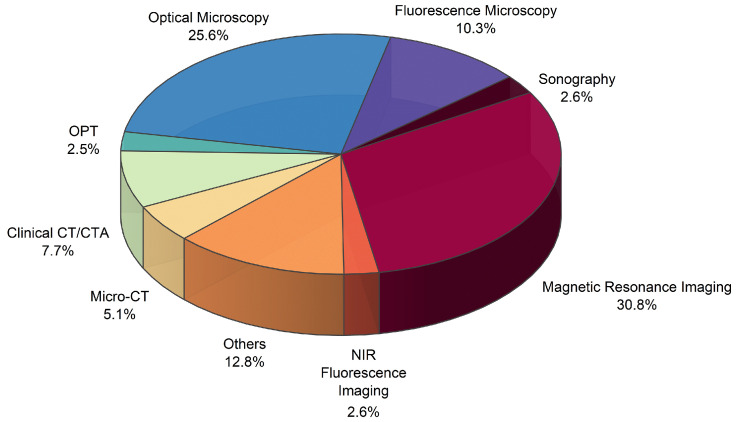
Imaging techniques present in the analyzed articles. CT: Computed Tomography; CTA: Clinical Tomography Angiography; OPT: Optical Projection Tomography; NIR: Near Infrared.

Regarding imaging techniques, micro-CT, clinical CT/CT angiography, optical microscopy tomography, optical and fluorescence microscopy, sonography, MRI, and NIR fluorescence, are present in this study. The majority of studies utilized MRI (30.8%) and optical microscopy (25.6%) to investigate the vascular network, while clinical CT, CT angiography, and micro-CT accounted for 12.8% of the articles (Figure 3). Other techniques include arthroscopy and second-harmonic-generation microscopy (Figure 3). MRI is the most commonly used technique, as the majority of the articles included in this review are clinical studies, where MRI is widely utilized for both diagnosis and pre-surgical evaluation.

**Figure 4:**
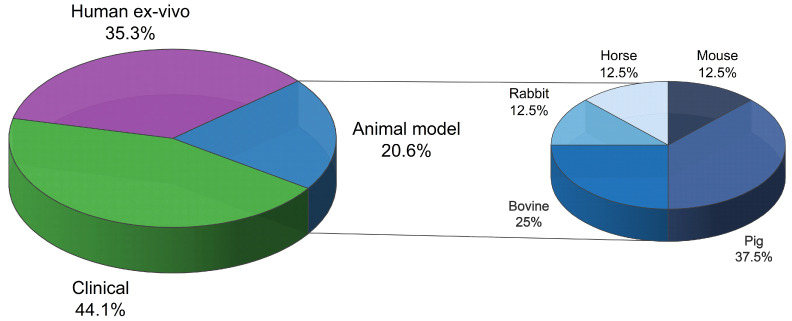
Distribution of the analyzed articles. The large pie chart on the left categorizes the articles by study type, with sections representing clinical, human ex vivo, and animal model studies. The small inset pie chart on the right specifies the animal species used. The study that utilized both bovines and rabbits has been counted twice in the animal species representation.

The analyzed studies were categorized into three groups: studies on animals, human samples, and human patients (Figure 4). As mentioned before, clinical studies are the majority (44.1%), followed by human ex vivo (35.3%), and animal studies (20.6%)(Figure 4). Various animal species, such as pigs, bovines, rabbits, horses, and mice, were included in this review (Figure 4). The majority of the animal studies focused on pigs and bovines, as these species are commonly used as models for meniscus research.

**Figure 5:**
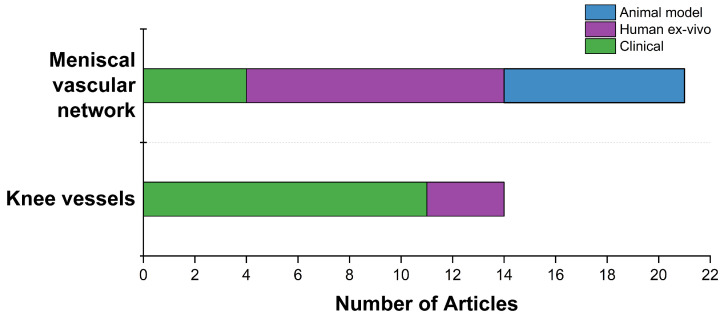
Comparison of the number of articles focused on the meniscal vascular network and knee vessels based on the study type. The knee vessels include the popliteal and genicular arteries.

A trend analysis of the blood vessels that were analyzed revealed that most articles focused on the meniscal vascular network, with a notable prevalence of human ex vivo studies. No articles involving animals examined the arteries that supply the meniscus whereas clinical studies often focused on knee arteries, indicating a strong interest among clinicians to further explore and clarify the position and structure of these arteries within the knee joint (Figure 5).

**Figure 6:**
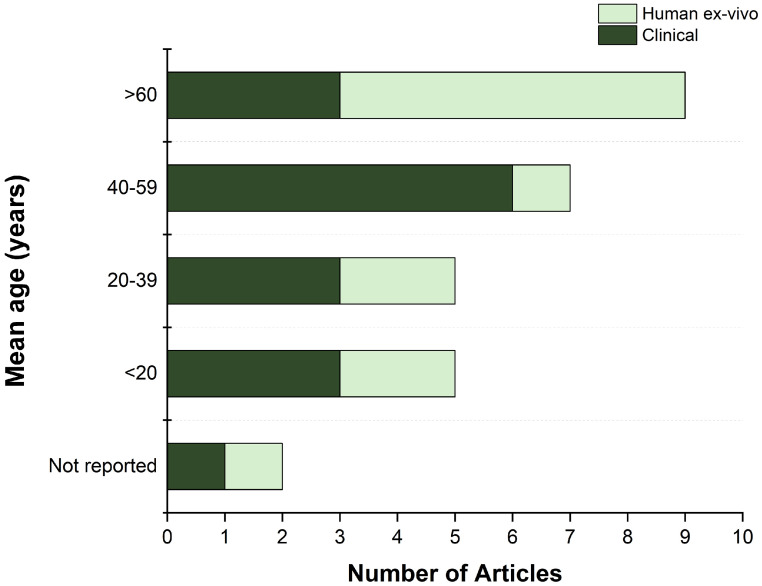
Distribution of mean ages in clinical and ex vivo human studies.

As the vascularization of the meniscus is age-dependent, the human studies were further classified into four groups based on the mean age of the analyzed cadavers or patients (Figure 6). Childhood and adolescence (<20 years), early adulthood (20–39 years), middle adulthood (40–59 years), and late adulthood (>60 years) age groups were defined. For two studies, the mean age was not reported. Most ex vivo studies examined old samples with a mean age over 60 years, primarily for the greater availability of cadavers from this age group. In contrast, the most common age group in clinical studies was middle adulthood (40–59 years), which is associated with a higher prevalence of knee and meniscal injuries (Figure 6).

Regarding the outcomes of interest, the imaging modalities used in the analyzed articles were evaluated for their diagnostic accuracy as well as their clinical and translational applications. Diagnostic accuracy was defined as the effectiveness of imaging techniques in identifying the meniscal vasculature and associated pathologies, whereas clinical and translational applications were defined, respectively, as the role of these imaging modalities in guiding clinical decisions, and contributing to the research and development of new therapies and the understanding of meniscal physiology and pathology.

In particular, clinical arthroscopy, combined with ICG fluorescence, allows for surface visualization of the meniscus and enhances vascular visualization during the procedure, enabling real-time perfusion assessment [25,38]. However, the injection of an ICG fluorescence agent into the bloodstream increases the invasiveness of the procedure. In contrast, noninvasive diffusion-weighted MRI can depict circulation and perfusion changes within the meniscus, distinguishing the zone-dependent blood supply of the meniscal tissue, while it offers a non-invasive alternative, it lacks the ability to provide high-resolution 3D vascular visualizations due to spatial resolution limitations [19]. Similar limitations were observed in ultrasound-based imaging techniques, which can detect major blood vessels but are unable to provide 3D, high-resolution representations of the meniscal vascular network [27]. Several studies employing traditional MRI have primarily focused on evaluating the anatomical positions and wall thickness of the popliteal and genicular vasculature within the knee joint [20,21,30,31,33]. Although MRI can guide treatment decisions and diagnostic assessment, it is limited in the ability to provide adequate visualization and quantitative evaluations of the meniscal microvasculature. To address this, contrastenhanced MRI was employed by Lin et al. to compare vascularity between neonatal and adult menisci [36]. Optical microscopy, commonly used in histological studies, is valuable for identifying and quantifying the meniscal microvasculature [7] and its arrangement with collagen fibers [9]. However, its limitation to 2D visualization prevents a complete representation of the meniscal vasculature. In contrast, 3D imaging techniques, such as 3D microscopy and micro-CT, are essential for revealing the complexity of the meniscal vascular network and provide the most accurate method for quantifying vascular density within each meniscal zone [10,15,16,40]. Despite their potential, only a few studies have employed 3D high-resolution imaging techniques for meniscal vascularization, underscoring the need for further research in this area.

The main limitations of this systematic review are potentially related to the research strategy. By focusing exclusively on studies published in the last 10 years, we may have excluded previous studies that provide valuable insights or foundational knowledge on the meniscal vascular network. However, focusing on recent literature allowed us to capture the latest advancements in imaging techniques, which are essential for understanding current capabilities in vascular imaging. Additionally, despite the careful consideration of potential keyword variants, the search string may have filtered out relevant articles due to differences in terminology or phrasing used across studies. Finally, while we selected primary databases for medical and scientific literature, future reviews could benefit from including a broader range of databases.

In conclusion, our review aims to encourage further investigation of the meniscal vascular network using 3D imaging techniques. By focusing on these advanced imaging modalities, researchers can elucidate the role of vascularization in meniscal health and pathology, and improve current surgical and treatment strategies.

## Figures and Tables

**Table 1:** Summary of the articles included in the review.

Clinical/Animal Model/Human Ex Vivo	Imaging Modality	2D or 3D Visualization	Imaging Resolution	Range of Vessel Size Detection	Evaluations	Main Results	Imaging Impact	Ref.
Clinical (*n* = 20 patients, range 49–84 y, mean 59.4 y)	Radiographic angiography	2D	Not reported	Arteries	Evaluate the effect of GA embolization on hypervascular GAs.	GA embolization was successfully performed in 100% of patients.	Radiographic Angiography reveals a hypervascular blush over the medial inferior aspect of the knee from the superior medial GA branches. Post-embolization radiographic angiography shows truncation of the hypervascular synovium.	Bagla et al., 2020 [23]
Clinical (*n* = 45 patients, mean 14.1 ± 2.2 y)	MRI	2D	3 mm slice thickness	Arteries	Analysis of the location of neurovascular structures. Define the risk of bicortical fixation.	The PA has a different risk of injury during tibial tubercle depending on the anatomical region.	The present study clarifies that the popliteal vessels are at risk of injury during tibial tubercle screw fixation.	Biolatto et al., 2023 [17]
Clinical (*n* = 18 patients, range 56–87, mean 68.6 ± 8.9 y)	MRI	2D	2–3 mm slice thickness	Arteries	Quantification of bone marrow lesions and meniscal injuries in relation to GA embolization.	Large bone marrow lesions and severe meniscal injuries on MRI indicated poor responses to GA embolization.	MRI is a useful tool to exclude potential nonresponders to GA embolization in advance.	Choi et al., 2020 [18]
Clinical (*n* = 126 patients, range 16–92 y, mean 58 y)	CT angiography	2D	5 × 5 mm slice thickness	Arteries	Measure the distance of the PA and the tibial plateau of normal and OA patients.	No significant correlation between PA to tibial plateau distance and OA grade. No significant correlation was seen in the PA to tibial plateau distances across age, gender, and racial origin. Any presence of aberrant anterior tibial artery.	CT angiography has the advantage of reproducibility, and reliability when measuring the PA distance without external compression. Disadvantages of CT angiography imaging stem from potential contrast media complications and radiation concerns.	Ezamin et al., 2017 [24]
Clinical (*n* = 50 patients, range 23–67 y, mean 41.2 ± 11.5 y)	Diffusion-weighted MRI	2D	0.20 × 0.59 × 4 mm and 1 × 1 × 4 mm	Arteries	Investigate if diffusion-weighted MRI imaging depicts microcirculation of meniscus. Evaluate the perfusion changes in meniscal disorder.	Among the normal, degenerated, and torn meniscus, the perfusion fraction is lower in the red zone of torn horns compared to the normal horns. Pseudo- and real-diffusion coefficients did not exhibit significant differences among different groups.	Provide valuable imaging information to guide the treatment of meniscal disease.	Guo et al., 2016 [19]
Clinical (information on patients not reported)	Arthroscopy (cyanine dye ICG fluorescence)	3D	Not reported	Arteries	Real-time evaluation of the vascularity of the meniscus and intra-articular apparatus before and after meniscal repair.	Evaluation of case-specific vascularity of the knee joint.	ICG fluorescence-guided arthroscopy enabled real-time evaluation of the vascularity of the knee structures, including the meniscus.	Kamimura, 2024 [25]
Clinical (*n* = 25 patients, range 16–56 y, mean 31.80 ± 11.55 y)	MRI	2D	Not reported	Arteries	Axial and sagittal plane studies were used to measure the shortest distance between the PA, popliteal vein, and tibial nerve to the posterior knee structures	During 90-degree knee flexion, neurovascular structures shift posterolaterally compared to the fully extended position. This movement occurs at the joint line level and 1 cm above and below the joint line.	MRI was used to investigate the location of the posterior neurovascular bundle in relation to the posterior aspect of the femur, tibia, and posterior cruciate ligament during both full knee extension and 90-degree flexion.	Keyurapan et al., 2016 [20]
Clinical (*n* = 97 patients, mean 25.3 ± 12.2 y)	MRI and arthroscopy	2D	4 mm intervals with 1.0 mm interception gap, 160 × 160 cm field of view, scan matrix 256 × 320	Arteries	Assess the orientation and distance of the PA from both the anteromedial and anterolateral portals. Location of PA during knee extension and flexion. Comparison of PA location in relation to damaged or intact anterior CL.	The figure-of-four knee position provides a safer distance from the posterior tibial cortex to the PA compared to full extension, especially for anterior CL-deficient knees. All-inside meniscus suturing from the anteromedial portal is safer in the figure-of-four position. Meniscus repairs should precede anterior CL reconstruction for optimal vascular safety and ease of access.	MRI data from unanesthetized individuals without fluid inflation likely show shorter distances between the posterior tibial cortex and the PA. Identification of the safe position of the PA for all-inside meniscus suturing.	Nishimura et al., 2015 [26]
Clinical (*n* = 100 patients, range 20–48 y, mean 36.3 y)	MRI	2D	3 mm slice thickness	Arteries	Evaluation of the inferior lateral GA diameter and its superoinferior position. Distance between the meniscocapsular junction and the inferior lateral GA.	The distance between the inferior lateral GA and meniscocapsular junction was significantly smaller in the middle zone than in the other three zones. Differential height of the inferior lateral GA in the different meniscal zones.	MRI offers advantages over cadaveric studies for evaluating small vessels like the inferior lateral GA, allowing for easy observation of tissue relations with scout navigation. Digital measurements provide accurate and reliable assessments of anatomical parameters.	Park et al., 2018 [21]
Clinical (*n* = 35 patients, range 18–66 y, mean age 41.6 y)	US	2D	Not reported	Arteries	Evaluation of remodeling of polyurethane meniscal implants post-reconstruction. Assessment of meniscal extrusion. Presence of vessels.	Mean values of extrusion in the supine position and during 90-degree flexion were significantly greater in the operated limb compared to the contralateral limb. No significant differences in extrusion were found between the limbs in a standing position.	US allowed the detection of the main blood vessels and serves as evidence of ongoing remodeling of blood vessels within the meniscal implants.	Poboży et al., 2023 [27]
Clinical (*n* = 144 patients, range 10–18 y, mean 14.5 ± 2.6 y)	MRI	2D	3 mm slice thickness	Arteries	Quantify the distance of the popliteal vasculature from various points of the posterior horn of the lateral meniscus. Quantify how this distance changes as a function of different patient characteristics.	The distance between the posterior horn of the lateral meniscus and the popliteal vasculature increases linearly throughout development. Significant associations between height, weight, body mass index, and skeletal maturity and these anatomic distances.	The evaluation of PA from meniscus through MRI helps in setting the appropriate meniscal suture penetration depth and reduces the risk of intraoperative vascular compromise during meniscal repairs.	Schachne et al., 2019 [22]
Clinical (*n* = 1 patient, 18 y)	MRI and Focused US-Doppler	2D	Not reported	Arteries	Intra-articular knee hemangiomas.	MRI showed a lobulated, multi-septate T2 hyperintense lesion with hypointense septae, suggesting a slow-flow vascular malformation in Hoffa’s fat pad. US-Doppler revealed small hypoechoic spaces with venous vascularity.	MRI, particularly T2-weighted images, is highly effective for detecting knee hemangiomas. The lesion shows a high signal on T2 images due to blood in vascular spaces.	Srampickal et al., 2017 [28]
Clinical (*n* = 8 patients, mean 64 ± 8 y)	NIRF imaging and Optical microscopy (IHC, anti-CD31 antibody)	3D and 2D	Not reported	Arteries and arteriole	Assess whether the vascularisation of the meniscus could be visualised intra operatively using NIRF imaging with ICG.	Meniscal vascularisation using NIRF imaging was observed in 75% of patients in whom vascularisation was demonstrated with IHC. The median extent of vascularisation was 13% using NIRF imaging and 15% using IHC.	This study shows the potential of NIRF imaging to visualize vascularisation of the meniscus, as vascularisation was observed in six out of eight patients with histologically proven meniscal vascularisation.	van Schie et al., 2022 [29]
Clinical (*n* = 170 patients, range 40–67 y, mean 52.2 ± 6.7)	MRI	2D	Partition thickness of 1.5 mm and an in-plane resolution of 0.31 × 0.31 mm.	Arteries	PA wall thickness.	PA wall thickness was positively associated with medial tibial cartilage loss over 2 years.	Vascular pathology negatively impacts knee cartilage. Targeting vascular pathology may be a potential target for osteoarthritis prevention.	Wang et al., 2015 [30]
Clinical (*n* = 176 patients, range 40–70 y, mean 56.5 y)	MRI	2D	0.16 × 0.16 × 2.5 mm	Arteries	Measurement of PA wall thickness.	Increased PA wall thickness was associated with structural progression in knee OA.	The findings support a role for vascular pathology in the progression of knee OA. Targeting atherosclerosis has the potential to improve outcomes in knee OA.	Wang et al., 2022 [31]
Human ex vivo (*n* = 24 knee joints, 50–70 y)	Light microscopy (histology)	2D	Olympus BX41 photomicroscope	Arteries, arteriole, and capillaries	Evaluate and compare the vascularity of the lateral and medial meniscus in male and female cadavers.	Distinctions in the distribution of blood vessels in male and female menisci and some differences in lateral and medial meniscus.	This work provided detailed information on the microstructure and composition of both lateral and medial meniscus in males and females. Highlights the detrimental effects of histological structure on different meniscal injuries.	Aggad et al., 2024 [7]
Human ex vivo (*n* = 12 knee joints, range 65–84 y, mean 71.4 y)	Clinical CT	3D	Reconstruction thickness 0.6 mm, reconstruction spacing 0.4 mm, and display field of view 170 mm	Arteries and arteriole	Evaluation of vascular perfusion. Characterization of the vascular pattern around the knee. Qualitative assessment of the small- and medium-sized vasculature.	Major blood vessels around the knee are at risk of injury during opening- and closing-wedge distal femoral and proximal tibial osteotomies, with medium-sized vessels near the osteotomy cuts being particularly vulnerable. GAs on the opposite side of the surgical field are at risk of injury during these procedures.	3D model from CT scan slices allowed the determination of distances between osteotomy cuts and vessels not necessarily at predetermined points.	Bisicchia et al., 2015 [32]
Human ex vivo (*n* = 6 menisci, range 9–18 y, mean 13.6 y)	Light microscopy (histology)	2D	10× Magnification Axioskop microscope, pixel size 1.02 μm	Capillaries	Evaluation of cellularity, arrangement of collagen fibers, and vascularity of discoid lateral meniscus.	No blood vessels were found in the inner part of discoid lateral meniscus in all patients but the 18-year-old. Endothelial cells, edematous tissue, and leaking of erythrocytes in the extracellular matrix were also observed in the specimen.	This work allowed the assessment of variation in terms of vascularity and disorganization of collagen fibers in the discoid lateral meniscus.	Bisicchia et al., 2018 [9]
Human ex vivo (*n* = 34 menisci, mean 21 ± 6.1 y)	3D light sheet microscopy (tissue clearing, anti-CD31 antibody) and Light microscopy (histology)	3D and 2D	Leica DMi8 fluorescence microscope, 40× of selected areas were taken with a Nikon Eclipse Ti-2 fluorescence microscope	Arteries, arteriole, and capillaries	Identification and characterization of the resident progenitor cell populations within the circumferential meniscal zones. 3D mapping of the meniscal vasculature related to the circumferential zones.	Resident mesenchymal progenitor cells were present in all 3 meniscal zones of healthy donors. Larger vessels in RR zone of meniscus were observed spanning toward RW zone sprouting to smaller arterioles and venules. Blood vessels were identified in all zones.	The presence of resident mesenchymal progenitors was evident in all 3 meniscal zones. 3D imaging demonstrated the presence of vascularization in WW zone of the meniscus.	Chahla et al., 2021 [10]
Human ex vivo (*n* = 240 knee joints, range 18–60 y, mean 37.3 ± 13.2 y)	MRI	2D	Not reported	Arteries	Assessing the risk of neurovascular injury and the danger zone in repairing the lateral meniscus.	The danger zone for lateral meniscus repair through the anteromedial portal extends 1.82 ± 1.68 mm laterally and 3.13 ± 2.45 mm medially from the patellar tendon. Through the anterolateral portal, the danger zone extends 2.81 ± 1.94 mm laterally and 1.39 ± 1.53 mm medially from the patellar tendon.	Surgeons can reduce the risk of iatrogenic neurovascular injury by avoiding the use of all-inside meniscal devices in the defined danger zone.	Chuaychoosakoon et al., 2021 [33]
Human ex vivo (*n* = 12 menisci, age not reported)	Immunofluorescence imaging (anti-CD31 antibody)	2D	Not reported	Capillaries	Investigate the role of blood vessels in the homeostasis of the meniscus.	Immune cells were mainly distributed around the blood vessels in the meniscus.	Variations in the meniscal microenvironment were found during degeneration. The study suggested that blood vessels play an important role in acute and chronic inflammation.	Fu et al., 2022 [34]
Human ex vivo (menisci from *n* = 15 patients, range 52–80 y, mean 66.9 y)	Optical microscopy and Immunofluorescence imaging (anti-EDG-1)	2D	200 nm	Arteries, arteriole, and capillaries	Histological and immunohistochemical evaluation of RA and OA menisci.	Distribution of endothelial cells in RA and OA menisci showed similar results.	Staining with anti-EDG-1 was performed to demonstrate the distribution of endothelial cells and served thus as the basis for the zonal division of the meniscus.	Fuhrmann et al., 2015 [35]
Human ex vivo Neonatal (*n* = 5 knee joints, 0–6 m) and adult (*n* = 5 knee joints, range 34-60 y, mean 50.2 y)	CE MRI	2D	0.4 × 0.4 × 1.0 mm	Arteries and arterioles	Assessment and comparison of vascularity in neonatal and adult specimens.	Neonatal menisci exhibited 6.0-fold greater overall post-contrast meniscal signal enhancement compared to adults, with significant perfusion in both peripheral and central zones Despite the similar distribution of perfusion between the lateral and medial meniscus, neonatal specimens showed greater blood flow overall.	Younger menisci receive a greater overall blood flow than adult menisci, including the central zone. This suggests that the immature meniscus is more biologically active than the adult meniscus.	Lin et al., 2020 [36]
Human ex vivo (*n* = 51 menisci, range 3–79 y, mean 25.6 ± 20.4 y)	Optical microscopy (histology)	2D	200 nm	Arteries, arteriole, and capillaries	Immunohistological staining in combination with serial sections and standardized software- based contrast detection. Color-channel quantitative analysis in 15 sections of each meniscus.	Microvascular density in the meniscus of the human knee decreased with increasing age (specifically in capsule and RR zones). No vessel formations were detected in the RW and WW zones after adolescence. The capsule is far more densely vascularized than any other part of the meniscus.	This study presents quantitative histological data on the microvascular anatomy of human knee menisci across a wide age range.	Michel et al., 2021 [12]
Human ex vivo (*n* = 6 menisci, range 62–86 y, mean 75 y)	CE CT	2D and 3D	range: 15–60 μm voxel size	Arteries and arteriole	3D meniscal vascular network visualization related to circumferential and radial zones. Quantitative analysis of vascular parameters (vascular volume, vascular length, diameter, tortuosity, and connectivity).	Highest vascular volume in peripheral PM zone 0 and zone 1. The mid-posterior radial portion showed the lowest contribution to the overall meniscal vasculature. The vascular segments of PM zone had a different diameter compared to the other circumferential zones.	Micro-CT permitted a quantitative analysis of vascular morphological parameters and topology. The study highlights the importance of spatial resolution in accurately characterizing the vascular network.	Orellana et al., 2024 [16]
Human ex vivo (*n* = 12 knee joints, range 68–81 y, mean 74.2 y)	Image Scanner on plastinated samples	2D	300 dpi	Arteries	Investigation of the saphenous nerve, its branches, and the PA to determine the optimal position for the posteromedial knee portal.	The PA is located 8.66 ± 2.17 mm dorsal to the joint capsule at the medial epicondyle level and 7.86 ± 2.26 mm from the posterior CL. At the tibial attachment of the posterior CL, the distance between the PA and the posterior CL is 5.93 ± 3.61 mm.	Plastination creates transparent body slices with intact structures and connective tissues and enables the production of transparent slices that can be scanned for morphometric analysis.	Sora et al., 2015 [37]
Human ex vivo (*n* = 10 knee joints, range 46–87 y, mean 66.4 ± 11.1)	Optical microscopy (histology)	2D	200 nm	Arteries, arterioles, and capillaries	Histological scoring and analysis of meniscus sections. Evaluation of the phenotypic and integrin expressions of meniscal cells in vascular and avascular areas.	Blood vessels are distributed along the “tree-like” transverse collagen fiber. The “tree-like” structure was more degenerate and without blood vessels in the Grade 3 menisci.	Provide an evaluation of cellular phenotypic changes and extracellular matrix alterations.	Wang et al., 2020 [13]
Animal model Bovine stifle joints (*n* = 6 stifle joints, 1.5–2.5 y)	Second harmonic generation microscopy	2D and 3D	Zeiss LSM 510 NLO laser-scanning multi-photon microscope with a 40× objective	Arteries, arteriole, and capillaries	3D organization of collagen fibers. Relationship between the fibers and blood vessels in the menisci.	Tie-fiber sheets surround the blood vessels and an associated proteoglycan-rich region. The size of tie-fiber sheets surrounding the vessels appeared to be associated with the size of the blood vessel.	The imaging technique used allowed the identification of a previously undescribed meniscal region surrounding the blood vessels.	Andrews et al., 2014 [14]
Animal model Bovine menisci (*n* = 2 menisci, age not reported), Rabbit menisci (*n* = 2 menisci, age not reported)	Optical microscopy and OPT	2D	Optical: 200 nm and OPT: 5–10 μm	Arteries, arteriole, blood vessels in the order of 10–100 μm	Analysis of the meniscus structure and tissue organization from the body to insertional roots.	Blood vessels were prevalent in the periphery of the root. These blood vessels then arborized to cover the anterior femoral surface of the meniscus	OPT was capable of imaging the entire structure of transition in the lapine meniscus.	Andrews et al., 2015 [8]
Animal model Pig (*n* = 1 stifle joint, 3 m)	Arthroscopy (ICG fluorescence)	3D	Not reported	Arteries	Assessment of the microvasculature within the healthy meniscus and in the medial meniscus after the induction of a radial tear in the middle-to-posterior section.	No fluorescence was detected in the meniscus with solutions diluted by 1000× and 100× and in the medial meniscus; Fluorescence was visualized at the synovium and anterior CL using ICG diluted by 10×.	The optimal dilution and dose setting of ICG for knee arthroscopy was 10×. The meniscus showed no active blood flow, even in the RR zone.	Kamimura, 2024 [38]
Animal model Neonatal (*n* = 10 menisci) and adult (*n* = 10 menisci, 3–4 y) porcine menisci	Micro-CT (Critical point drying) and Optical microscopy (immunohistochemistry, anti-CD31 antibody)	2D and 3D	3.3 μm voxel size	Arteries, arteriole, and capillaries	3D meniscal vascular network visualization. Quantitative analysis of vascular parameters (vascular volume, vascular length, thickness, tortuosity, and connectivity).	Higher number of blood vessels, more branching, and higher tortuosity in neonatal compared to adult menisci. Adult menisci have fewer and thicker vascularity.	Micro-CT imaging allowed detailed quantitative volumetric analysis of vascularized meniscal structures in porcine neonatal and adult menisci.	Karjalainen et al., 2024 [15]
Animal model Horses (*n* = 15 stifle joints, 0–30 y)	Optical microscopy	2D	200 nm	Arteries, arteriole, and capillaries	Presence of blood vessels in relation to age and to the circumferential zone of the meniscus	Age-dependent extent of vascular supply. In young animals, blood vessels marginally reached the middle (RW) zone. In older horses (>5 years), only the RR zone was vascularized.	The histologic architecture of equine menisci closely resembles that of humans. Age-dependent decrease in vascularization in older horses.	Kremer et al., 2017 [11]
Animal model Guinea pigs (*n* = 6 pigs, 8–10 and 17–19 m)	3D microscopy of fluorophores	3D	Not reported	Arteries	Visualization of tracer transport (Texas-red and Rhodamine-green) in serial-sectioned, cryofixed block specimens.	Texas-red tracer was abundant in the marrow cavity albeit less prevalent or absent in the bone, cartilage, meniscus, and other tissues of the joint. Tissues of the meniscus, ligament, and tendon exhibited abundant Rhodamine-green tracer, volumes of tissue containing this molecular tracer were significantly lower in older than in younger animals.	3D microscopy of fluorophores allowed a new understanding of joint physiology, the role of intra-articular and extra-articular transport of inflammatory cytokines on tissue permeability, and physical and chemical measures to modulate joint tissue health with age.	Ngo et al., 2018 [39]
Animal model Transgenic mice (*n* = 6 menisci, 6 m, endothelial cells were induced to emit red fluorescence)	3D light sheet microscopy (tissue clearing)	3D	4× magnification, detector pixel size 6.5 μm	Arteries, arteriole, and capillaries	3D meniscal vascular network visualization. Quantification of vascular volume.	No significant differences between the circumferential meniscal zones, Indication of having new circumferential classification. The anterior region had the most abundant blood supply.	Tissue clearing allowed 3D imaging of the meniscal blood supply and quantitative vessel distribution.	Sheng et al., 2023 [40]

CE = Contrast-Enhanced; CL = Cruciate Ligament; CT = Computed Tomography; GA = Genicular Artery; ICG = Indocyanine Green; IHC = Immunohistochemistry; MRI = Magnetic Resonance Imaging; NIRF = Near-Infrared Fluorescence; OA = Osteoarthritis; OPT = Optical Projection Tomography; PA = Popliteal Artery; PM = Perimeniscal; RA = Rheumatoid Arthritis; RR = Red-Red; RW = Red-White; US = Ultrasound; WW = White-White.

## Data Availability

No new data were created or analyzed in this study. Data sharing is not applicable to this article.

## Abbreviations

The following abbreviations are used in this manuscript:

2D	Two-dimensional
3D	Three-dimensional
ACL	Anterior Cruciate Ligament
C	Comparison
CECT	Contrast-Enhanced Computed Tomography
CTA	Computed Tomography Angiography
I	Intervention
ICG	Indocyanine Green
Micro-CT	Micro-Computed Tomography
MRI	Magnetic Resonance Imaging
NIR	Near-Infrared
O	Outcome
OA	Osteoarthritis
P	Population
PCP	Perimeniscal Capillary Plexus
PGs	Proteoglycans
PM	Perimeniscal
PRISMA	Preferred Reporting Items for Systematic Reviews and Meta-Analyses
RR	Red-Red
RW	Red-White
WW	White-White
